# Olfactory Training in Patients with Parkinson's Disease

**DOI:** 10.1371/journal.pone.0061680

**Published:** 2013-04-17

**Authors:** Antje Haehner, Clara Tosch, Martin Wolz, Lisa Klingelhoefer, Mareike Fauser, Alexander Storch, Heinz Reichmann, Thomas Hummel

**Affiliations:** 1 Smell & Taste Clinic, Department of Otorhinolaryngology, University of Dresden Medical School, Dresden, Germany; 2 Division of Neurodegenerative Diseases, Department of Neurology, Dresden University of Technology, Dresden, Germany; 3 Department of Neurology, University of Dresden Medical School, Dresden, Germany; 4 Department of Neurology, Elblandkliniken Meissen, Meissen, Germany; Duke University, United States of America

## Abstract

**Objective:**

Decrease of olfactory function in Parkinson's disease (PD) is a well-investigated fact. Studies indicate that pharmacological treatment of PD fails to restore olfactory function in PD patients. The aim of this investigation was whether patients with PD would benefit from “training” with odors in terms of an improvement of their general olfactory function. It has been hypothesized that olfactory training should produce both an improved sensitivity towards the odors used in the training process and an overall increase of olfactory function.

**Methods:**

We recruited 70 subjects with PD and olfactory loss into this single-center, prospective, controlled non-blinded study. Thirty-five patients were assigned to the olfactory training group and 35 subjects to the control group (no training). Olfactory training was performed over a period of 12 weeks while patients exposed themselves twice daily to four odors (phenyl ethyl alcohol: rose, eucalyptol: eucalyptus, citronellal: lemon, and eugenol: cloves). Olfactory testing was performed before and after training using the “Sniffin' Sticks” (thresholds for phenyl ethyl alcohol, tests for odor discrimination, and odor identification) in addition to threshold tests for the odors used in the training process.

**Results:**

Compared to baseline, trained PD patients experienced a significant increase in their olfactory function, which was observed for the Sniffin' Sticks test score and for thresholds for the odors used in the training process. Olfactory function was unchanged in PD patients who did not perform olfactory training.

**Conclusion:**

The present results indicate that olfactory training may increase olfactory sensitivity in PD patients.

## Introduction

Impairment of olfaction is a characteristic and early feature of Parkinson's disease (PD). Recent data indicate that >95% of patients with PD present with significant loss of olfactory function [1] whose impact on daily life is often underappreciated [2]. The sense of smell makes a significant contribution to the quality of life and the ability to experience pleasure. According to a study by Politis et al. [3] olfactory loss belongs to the top-five most prevalent motor and non-motor symptoms in early stage PD patients that affect their quality of life. Only pain is referred to as a more prevalent troublesome non-motor problem in this patient group. Specifically, patients with impaired olfaction are more likely to experience depressive symptoms as they express severe limitations in relation to the enjoyment of food and drinks and socializing [4], [5].

Despite of the high prevalence of olfactory impairment in PD which even exceeds the prevalence of tremor ^1^ no therapy has yet been proven to be effective in PD-related smell loss. Studies indicate that pharmacological treatment of PD fails to restore olfactory function in PD patients [6], [7]. While appropriate investigations in larger groups of PD patients with olfactory function appear to be missing, dopamine agonists do not appear to have a significant effect on olfactory function in PD [8]. Recently, however, deep brain stimulation (DBS) has been added to the therapeutic armamentarium in PD. The study by Hummel et al. [9] indicates that DBS in PD patients improves odor discrimination while it has no effect on odor thresholds. These results are partly confirmed by a study in 15 patients [10] and a case report [11] where the identification of odors was found to become more precise during the ON period of the stimulator. Understandably, this therapeutic approach cannot be applied on a routine basis. Results are however consistent with previous studies suggesting that the olfactory sense has the ability to change and recover. In this context olfactory training has been shown to improve olfactory function in humans [12]–[14] and is now an acknowledged therapy in postinfectious and posttraumatic smell loss.

The goal of this single center, prospective, controlled, non-blinded study was to investigate the change of olfactory function in PD patients following olfactory training for 12 weeks consisting of frequent short-term exposure to various odors.

## Materials and Methods

### Subjects

Thirty five training subjects were recruited consecutively between May 2010 and August 2011 and 35 control subjects between May 2009 and May 2010 at the Division of Neurodegenerative Diseases, Department of Neurology at Dresden University of Technology. The control subjects participated in a longitudinal olfactory study. Eligible subjects were 18 years of age or older, had received the diagnoses of PD according to UK Brain Bank criteria [15], and were on stable anti-parkinsonian medication for at least 4 weeks prior to study enrollment and during the study. The following exclusion criteria had been defined: Identifiable cause of parkinsonism or signs for atypical parkinsonian disorders, dementia, and psychiatric conditions interfering with study participation.

Detailed information about the experiment was given to all participants and written consent was obtained. All aspects of the study were performed in accordance with the Declaration of Helsinki. The study protocol was approved by the local Ethics Board of the Faculty of Medicine of Dresden University of Technology.

### Training with Odorants

The training group performed olfactory training over a period of 12 weeks. Patients exposed themselves twice daily to four odors (phenyl ethyl alcohol (PEA): rose, eucalyptol: eucalyptus, citronellal: lemon, and eugenol: cloves). These four odorants were chosen to be representative of four odor categories claimed by Henning [16] in his work on the “odor prism” (*Geruchsprisma*), where he tried to identify primary odors (compare [17]). These categories are flowery: *blumig* (e.g., rose), foul: *faulig*, fruity: *fruchtig* (e.g., lemon), aromatic: *würzig* (e.g., cloves), burnt: *brenzlig*, and resinous: *harzig* (e.g., eucalyptus). Training patients received four brown glass jars (total volume 50 mL) with one of the four odors in each (1 mL each, soaked in cotton pads to prevent spilling). All jars were labeled with the odor name. Patients were asked to sniff the odors in the morning and in the evening for approximately 10 seconds each. To focus their attention on the training, they were asked to keep a diary in which they rated their overall olfactory abilities each Sunday (data not analyzed). Further, patients received a phone call by one of the experimenters 4 weeks after the training started (1) to ask about the patients' olfactory function and (2) to maintain compliance with the training procedure. Patients in the non-training group were advised to wait and see how the olfactory function would change.

### Olfactory Testing

Olfactory testing was performed before and after the training period of 12 weeks using the “Sniffin' Sticks” test kit [18] which involves tests for odor threshold, odor discrimination, and odor identification. Using commercially available felt-tip pens, the odorants were presented approximately 2 cm in front of both nostrils for 2 seconds. PEA odor threshold was assessed by a single-staircase, 3-alternative forced choice (3-AFC) procedure. Three pens were presented to the patient in a randomized order, two contained odorless solvent (propylene glycol) and the other an odorant in a certain dilution. The patient's task was to indicate the pen with the odorant. Concentration was increased if one of the blanks was chosen and decreased if the correct pen was identified twice in a row. The mean of the last 4 of a total of 7 reversal points was used as detection threshold (ranging from 1 to 16). A total of 16 odor concentrations were tested starting from a 4% stock solution (dilution ratio 1∶2; solvent propylene glycol). The second subtest assessed the ability of the patient to discriminate different odors. Again, 16 triplets of pens were offered, each including two identical odors and a different one. The patient's task was to indicate the pen which had a different smell. The score was the sum of correct responses ranging from 0 to 16. Both threshold and discrimination testing was performed with the patient being blindfolded. For testing of odor identification, 16 pens containing common odors were offered. The patient had to identify each of the odorants from a list of four descriptors. The sum of the scores from the three subtests resulted in the TDI-score (Threshold, Discrimination, Identification) with a maximum of 48 points. A score of 30.5 points or more indicates normosmia, a score between 16.5 and 30 points indicates reduced olfactory function in terms of hyposmia, and a score of less than 16.5 points indicates functional anosmia.

### Threshold Measures

While thresholds for PEA were measured using the single-staircase paradigm within the Sniffin' Sticks test kit (see previously discussed data), thresholds for the other odorants used for training (eucalyptus, eugenol, and citronellal) were assessed by means of the method of ascending limits [19], using a 3-AFC procedure. This procedure was chosen because it is slightly faster than the staircase procedure, although it may be somewhat less reliable [20]. Odors were presented in brown glass jars, similar to the presentation of PEA using the “Sniffin' Sticks”. Two of the jars contained odorless solvent (propylene glycol), the other an odorant in a certain concentration. The patient's task was to indicate the jar with the odorant. Correct identification was assumed when the patient correctly identified the same odor concentration three times in a row. A total of eight odor concentrations for each odor were tested starting from 4% stock solutions (dilution ratio 1∶4; solvent propylene glycol). Between tests of the odorants, subjects rested for approximately 5 minutes to minimize adaptation.

### Statistical analyses

Data were analyzed by means of SPSS 19.0 (SPSS Inc., Chicago, Ill, USA). If not mentioned otherwise, all data are displayed as means±standard deviation (SD) or numbers (%), significance level was set at p<0.05 (two-tailed test). Bonferroni tests were used for post-hoc analyses. Pearson statistics were used for correlational analyses.

## Results

### Study population

Thirty-five patients (18 men, 17 women; mean±SD age: 63.1±8.2 yrs (range: 43–76 yrs); mean±SD disease duration; 7.5±4.8 yrs; median Hoehn & Yahr stage: 2 (range: 1–4)) participated in the **“training group**”. In the **“control group**” 35 patients participated (27 men, 8 women; mean±SD age: 61.5±9.5 yrs (range: 45–76 yrs); mean±SD disease duration: 3.8±3.1 yrs; median Hoehn & Yahr stage: 1.9 (range: 1–3)). Descriptive statistics of the patient groups are shown in Table 1.

**Table 1:** Descriptive statistics of patients groups.

	Training group (n = 35)	Control group (n = 35)	p-value^a^
**Age (years)**	63.1±8.3	61.5±9.5	0.10
**Sex (n)**	17 **♀**, 18♂	8 **♀**, 27♂	0,03
**Duration of disease (years)**	7.5±4.8	3.8±3.1	0.001
**Hoehn & Yahr score** b	2.0 1–4	1.9 [1]–[3]	0.52
**TDI_t = 0_ score**	18.1±5.3	17.8±5.2	0.89
**threshold _t = 0_ score**	2.4±1.9	2.8±2.3	0.55
**discrimination _t = 0_ score**	8.1±2.7	7.0±2.6	0.31
**identification _t = 0_**	7.6±2.7	7.8±2.2	0.72
**TDI _t = 12 weeks_ score**	20.5±5.4	17.2±4.8	0.012
**threshold _t = 12 weeks_ score**	2.9±2.3	2.6±2.1	0.67
**discrimination_t = 12 weeks_ score**	9.2±2.3	6.6±2.5	0.001
**identification _t = 12 weeks_ score**	8.6±2.4	7.7±1.9	0.12

a p-values are from unpaired two-sided t-tests.

b Median [range].

### “Training group”

#### Psychophysics

**Figure 1:**
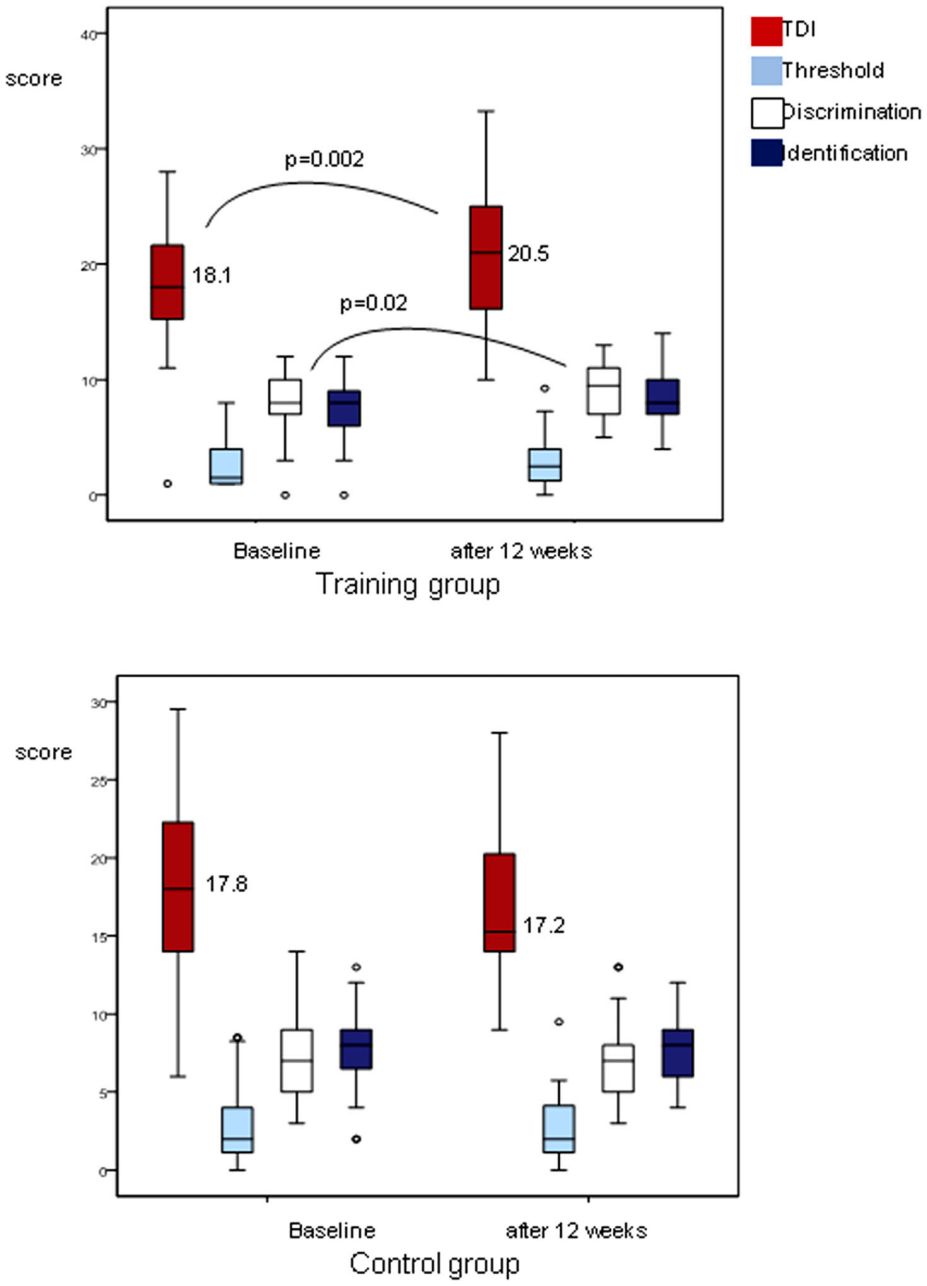
Olfactory function as expressed by the TDI score (comprehensive score of threshold, discrimination, and identification abilities) at baseline and after 12 weeks in the training group and the control group without training. Higher scores express higher olfactory sensitivity.

**Figure 2:**
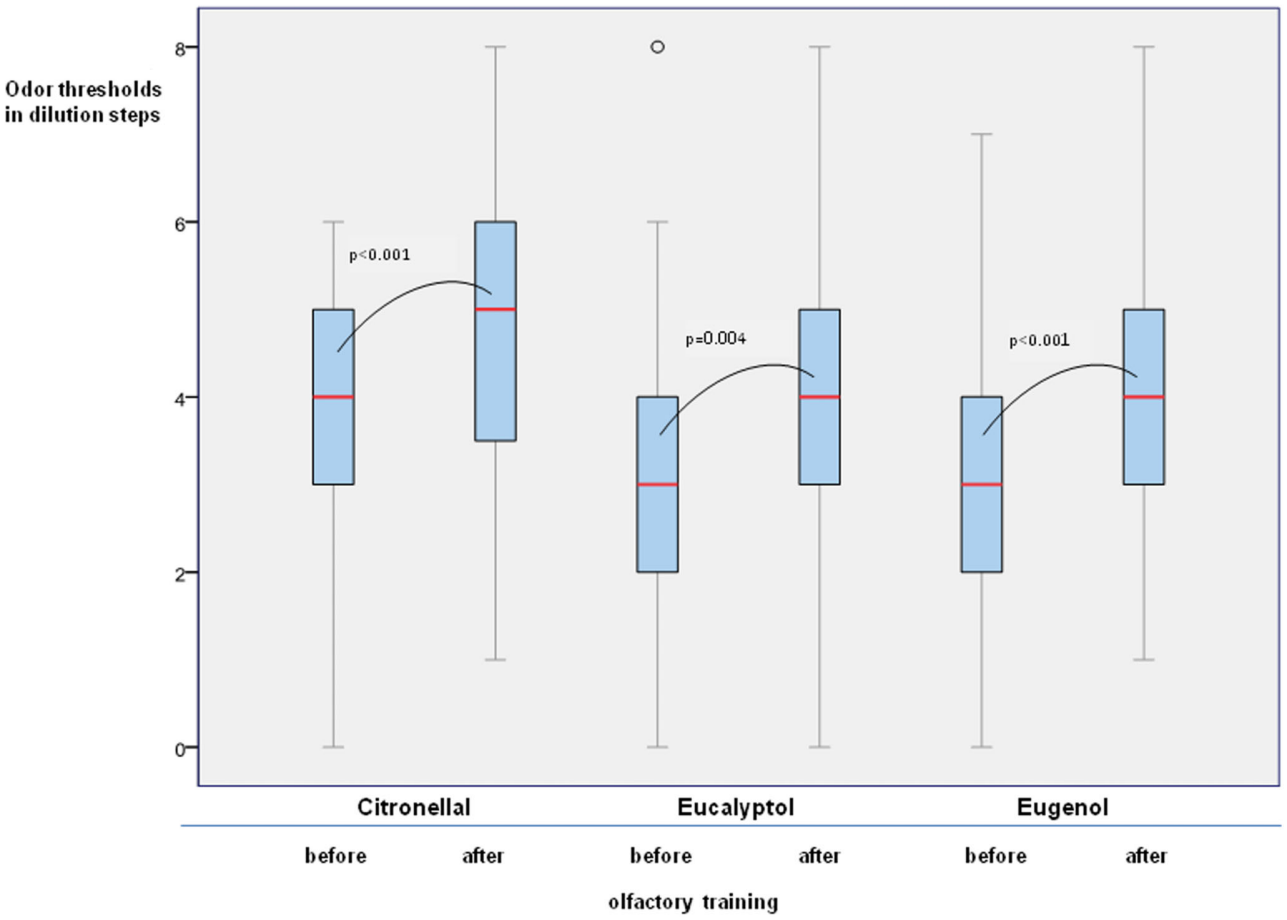
Threshold testing of the training odors citronellal, eucalyptol and eugenol before and after olfactory training. Higher odor thresholds express higher olfactory sensitivity.

Olfactory function expressed as TDI score was significantly different between baseline and after 12 weeks of training (t(35) = −3.37, p = 0.002; Figure 1). With regard to individual tests of olfactory function there was an effect of the factors “olfactory training” (F(1,34) = 6.67, p = 0.014), and “olfactory test” (F(2,32) = 125.26, p = 0.001) indicating that the training effect was reflected by the subtests in a different way. This was also indicated by *post-hoc* testing with paired two-sided t-tests which revealed that the groups differed for odor discrimination (t(35) = 2.5, p = 0.016), and that the groups tended to be different for odor identification (t(35) = 1.9, p = 0.065); no group difference was present for odor threshold (t(35) = 1.2, p = 0.24; Figure 1). In addition, thresholds for the other odorants used for training (eucalyptus, eugenol, and citronellal) improved significantly after the training period: eucalyptus t(35) = 3.05, p = 0.004; eugenol t(35) = 4.3, p = 0.001); citronellal t(35) = 3.97, p = 0.001 (Figure 2).

#### Sex-and age-related differences in olfactory improvement

Furthermore, when comparing TDI score difference between male and female PD patients, no significance was seen (t = 0.98, p = 0.34). Correlational analyses between TDI score difference_(baseline-12 weeks)_ in relation to the age of the patients revealed no significant correlations (r = 0.02, p = 0.94).

#### Relationship between olfactory improvement and severity/duration of disease

Correlational analyses between TDI score difference_(baseline-12 weeks)_ in relation to the severity of PD were made across all patients and, separately, for hyposmic patients only. However, there were no significant correlations (Pearson) between the TDI score difference, duration of disease (r = −0.18, p = 0.92), Hoehn & Yahr score (r = −0.07, p = 0.69), the UPDRS-III score (r = −0.01, p = 0.95) and the UPDRS-III score difference_(baseline-12 weeks)_ (r = −0.06, p = 0.76), respectively. Duration of the disease correlated significantly with Hoehn & Yahr score (r = 0.48, p = 0.001). When comparing UPDRS-III score before and after olfactory training no significant difference was found (t = 0.98, p = 0.34).

#### Differences between PD subtypes

With regard to the olfactory improvement there were significant differences between patients with different disease subtypes (tremor dominant type (n = 6), akinetic-rigid type (n = 13) or mixed type (n = 16) (F(2,32) = 4.46, p = 0.02). Hereby, the olfactory improvement was largest in tremor dominant type PD patients although baseline TDI scores proved not to be different between the groups (p = 0.8).

### Controls

#### Psychophysics

When assessed by means of the “Sniffin'Sticks” birhinal olfactory performance was not significantly different between baseline and after 12 weeks (TDI: t(35) = 0.14, p = 0.891; threshold: t(35) = −0.60, p = 0.55; discrimination: t(35) = 1.03, p = 0.31; identification: t(35) = 0.367, p = 0.72; Figure 1).

### “Training group” vs Controls

#### Psychophysics improvement

**Figure 3:**
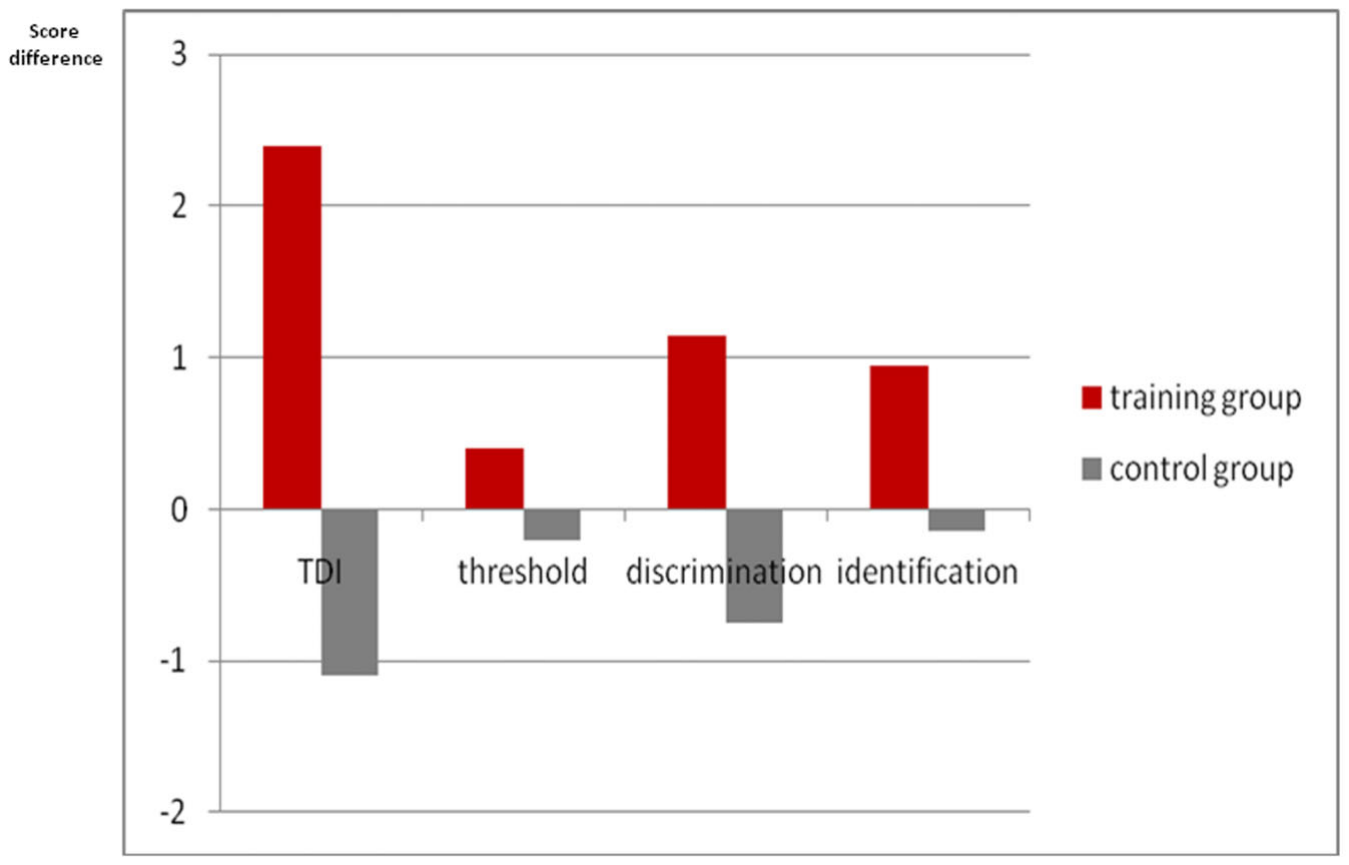
Change of olfactory function after 12 weeks as expressed by the TDI score (comprehensive score of threshold, discrimination, and identification abilities) in the training group compared to controls without training.

**Figure 4:**
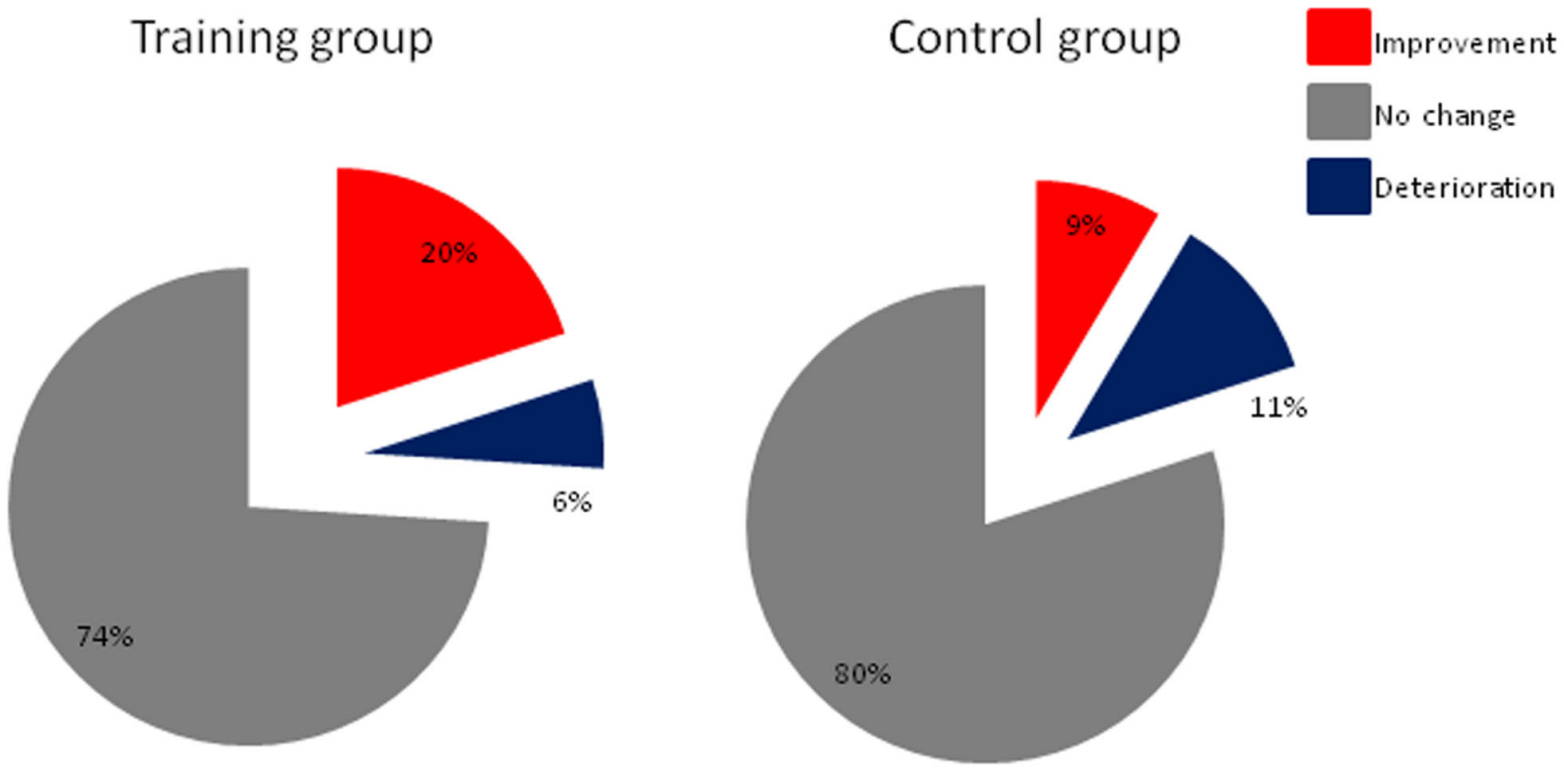
Change of olfactory function of more than 5.5 points in the TDI score (individual improvement).

When comparing TDI score differences between baseline and after 12 weeks between controls and the training group the latter group performed significantly better (F(1,68) = 10.41, p = 0.005). With regard to individual subtests only odor discrimination was significantly different between the two groups (F(1,68) = 10.25, p = 0.002), but not odor threshold (F(1,68) = 0.74, p = 0.34) and odor identification (F(1,68) = 20.63, p = 0.09; Figure 3). Furthermore, with regard to improvement on an individual level [21], seven of 35 subjects from the training group (20%) exhibited improvement of more than 5.5 points in the TDI score, whereas only 3 of 35 subjects exhibited improvement in the control group (9%) (Figure 4). Two of the patients in the training group (6%) exhibited a decline of olfactory function whereas this was the case in 4 patients (11%) in the control group.

## Discussion

Our results indicated that **A)** olfactory training produces both an improved sensitivity towards the odors used in the training process and an overall increase of olfactory function. It appears to increase olfactory function in 20% of the subjects over a period of 12 weeks compared to 9% of subjects who had no olfactory training. **(B)** Olfactory training proves to be useful independently from age, sex, duration and severity of the disease, and severity of olfactory dysfunction. **(C)** The training effect appears to be more pronounced in patients with tremor dominant type of PD.

These findings are consistent with the results of previous studies suggesting that the olfactory sense has the ability to change and recover. Such plasticity has been shown in animals e.g., for the odorant androstenone [22], a five odorant identification task [23], and comprehensive odorant detection training [24]. In the latter study, odorant-guided operant conditioning training proved sufficient to restore olfactory detection performance in cadmium-exposed mice with damaged olfactory function. Furthermore, repeated exposure of human subjects to androstenone [25], which has been shown by means of psychophysical and electrophysiological techniques supported the existence of plasticity in the peripheral olfactory system which may be reflected by an increased growth of olfactory receptor neurons and/or an increased expression of olfactory receptors in response to the exposure.

It is interesting to note that odor discrimination, but not odor threshold, improved in response to olfactory training. An explanation could be that odor discrimination appears to involve higher-level cognitive functions compared to odor thresholds (e.g., [26], [27]), especially as odor memory is intimately involved in the non-verbal odor discrimination paradigm. Support for our results comes from animal research [28] where rats were given extensive training with overlapping complex odorant mixtures and consequently showed improved behavioral discrimination abilities. Therefore, it might be hypothesized that olfactory training has positive effects on cognitive processing of olfactory information. Likewise, the observed effect may reflect improved attention to odors induced by the intense focus on odors during the training period. Motor function however, as reflected by the UPDRS motor score remains unchanged.

In our study we observed an age- and sex-independent response to olfactory training which was also not influenced by the duration and the severity of the disease. Consequently, PD patients equally qualify for olfactory training. With regard to the different disease subtypes however, tremor dominant PD patients had the greatest benefit from training. Katzen et al. [29] and Oh et al. [30] reported that PD patients with tremor-dominant subtype had the best cognitive function state and showed superior performance on overall cognitive function tasks, including language, memory ability and execution function. Considering the proposed close association between cognitive and olfactory functioning it might be hypothesized that the baseline cognitive state is determining the olfactory outcome. On the other hand, however, in terms of general olfactory function there are no major differences between subtypes of PD, namely tremor-dominant PD, akinetic-rigid PD, and equivalent-type PD [1].

Although our results seem to suggest that olfactory training may be helpful in PD patients with olfactory loss, they also raise numerous questions. One limitation of our study is that we used a non-blinded design. Future blinded studies need to determine 1) whether the observed increase of olfactory sensitivity is temporary or would stay even after the training period is over; 2) whether patients need to train with odors, or whether sniffing alone leads to the same results; 3) whether training with odors increases cognitive function of PD patients; and 4) whether training leads to an increase of the volume of the olfactory bulb or the responsiveness to odors at the level of the olfactory epithelium. These future studies will also have to use more balanced groups in terms of the disease subtype.

In conclusion, our results indicate that the structured, short-term exposure to odors may increase olfactory sensitivity in PD patients.
